# Impact of a Digital Balancing Tool on Femur and Tibial First Total Knee Arthroplasty: A Prospective Nonrandomized Controlled Trial

**DOI:** 10.1016/j.artd.2022.06.020

**Published:** 2022-09-22

**Authors:** Jan A. Koenig, Edgar A. Wakelin, Brandon Passano, Sami Shalhoub, Christopher Plaskos

**Affiliations:** a Department of Orthopedic Surgery, NYU Langone Hospital Long Island, Mineola, NY, USA; b Corin USA, Raynham, MA, USA

**Keywords:** Total knee arthroplasty, Predictive balance, Gap balancing, Accuracy, Outcome, Tibia first

## Abstract

**Background:**

Recent developments in intra-operative sensor technology provide surgeons with predictive and real-time feedback on joint balance. It remains unknown, however, whether these technologies are better suited to femur-first or tibia-first workflows. This study investigates the balance accuracy, precision and early patient outcomes between the femur-first and tibial-first workflows using a digital gap-balancing tool.

**Methods:**

One-hundred six patients had posterior cruciate ligament sacrificing total knee arthroplasty using a digital joint tensioner. The participants were divided into 4 groups with different visibility to balance data 1) Femur-first blinded data, 2) Femur-first not blinded data, 3) Tibia-first blinded data, 4) Tibia-first not blinded data with predictive balancing. Knee Injury and Osteoarthritis Outcome Score and University of California at Los Angeles activity level were recorded at 1-year.

**Results:**

Group 4 reported less midflexion imbalance (40°) compared to all other groups (1: 1.5 mm, 2: 1.7 mm, 3: 1.6 mm, 4: 1.0 mm, *P* < .031) and reduced variance compared to all other groups at 40° and 90° (*P* < .012), resulting in an increased frequency of joints balanced within 2 mm throughout flexion in group 4 (1: 69%, 2: 65%, 3: 67%, 4: 91%, *P* < .006). No differences were found between 3-month, 6-month, or 1-year outcome scores between technique.

**Conclusions:**

Improvements in balance were observed in midflexion instability and balance variability throughout flexion when a tibia-first approach in combination with a digital balancing tool was used. The combination of a digital balancing tool and a tibia-first approach allowed a target joint balance to be achieved more accurately compared to a non-sensor augmented or femur-first approach.

## Introduction

Achieving proper fit, fixation, and optimal joint balance are primary goals in total knee arthroplasty (TKA). Symptoms of poor joint balance, such as instability, stiffness, and pain, are some of the major causes of revision surgery [1,2]. Improving the ability to achieve a desired joint balance, therefore, may be instrumental in reducing revisions and improving outcomes in TKA.

Femur-first (FF) and tibia-first (TF) approaches in TKA achieve balance using different methods [3]. FF involves all bone resections to the femur in a prescribed way, before achieving joint balance by performing soft tissue releases (as in measured resection), recutting, downsizing the femur, or modifying the single plane of the tibial resection (as in kinematic alignment). TF gap balancing however applies a prescribed bone resection to the tibia before modifying the coronal and rotational position of the femoral component to achieve balance in the extension and flexion space independently.

New technology exists for measuring and predicting postoperative joint gaps [4,5]. However, there have been no studies investigating the ability of a surgeon to achieve a target joint balance using both FF and TF techniques with and without a digital joint tensioning device. In this study, we prospectively investigate the sequential impact of modifying surgical technique from FF to TF and introduction of a digital joint tensioning tool on the accuracy and precision of postoperative joint balance. This sequence modification allows the study to assess differing degrees of predictive, as opposed to reactive, intraoperative gap balancing. A secondary aim is to investigate any impact on early patient-reported outcomes. We hypothesize that the use of a predictive digital gap balancing tool with a TF approach will result in improved joint balance and that knees with improved joint balance will report improved outcomes.

## Material and methods

### Patient enrollment

A prospective investigation was performed by a single surgeon between March 2018 and June 2020. Institutional review board approval was obtained from New England Institutional Review Board. Inclusion criteria were end-stage osteoarthritis and scheduled for primary TKA. Exclusion criteria were body mass index >40 kg/m^2^, cancer, inability to complete outcome questionnaires, and joint infection.

### TKA technique and groups

**Figure 1:**
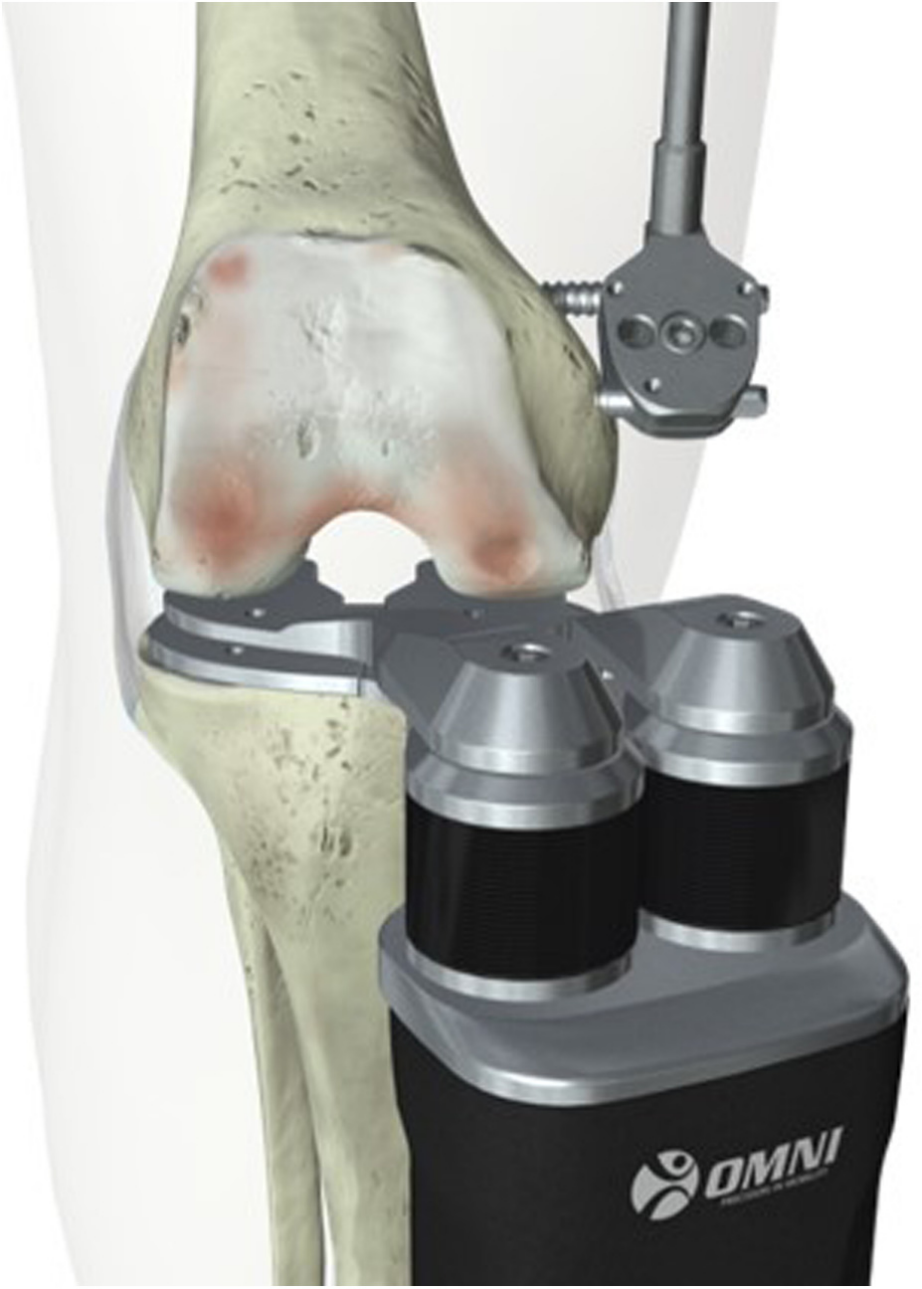
Digital balancing device used in this study, shown here tensioning the joint after the tibial resection for a predictive joint balance tibia-first workflow.

**Figure 2:**
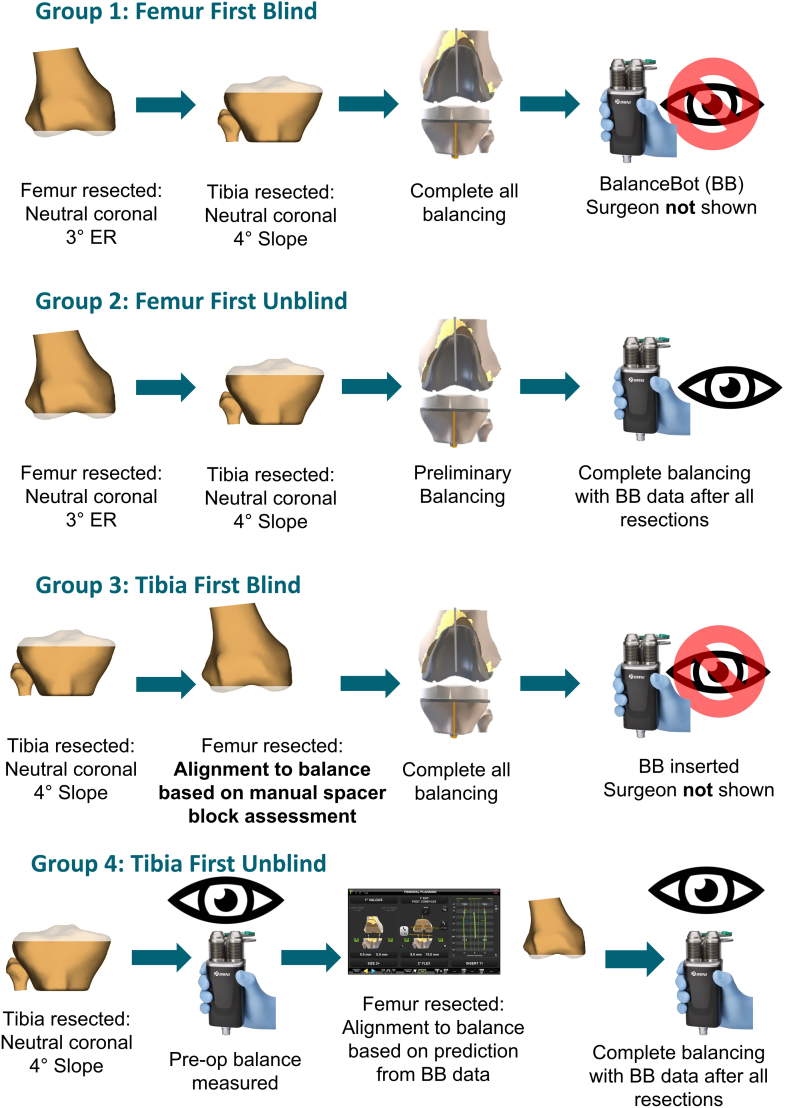
Description of study group surgical workflows showing sequential change in TKA technique from FF to TF and introduction of a digital joint balancing tool. ER, external rotation.

Prior to this investigation, the author/surgeon had nearly 10 years of experience using the OMNIBotics (Corin, UK) robot-assisted TKA platform with an FF measured resection approach and had not performed TF surgery with robotic assistance. In this study, a robotic joint tensioning tool integrated with the OMNIBotics system, the BalanceBot (Corin, UK), was introduced (Fig. 1). The BalanceBot provides a prescribed force (70-90 N per side) to the joint through the range of motion and measures the resulting medial and lateral joint gaps. When capturing data, the surgeon supports the thigh posteriorly in flexion and extends the knee taking care not to apply any external coronal or axial rotation. The surgeon then lowers the thigh into extension such that at terminal extension, the leg is only supported by the heel with the BalanceBot in place. The BalanceBot was used in 1 of 2 ways. First, it was introduced after all resections in an FF workflow, immediately before cementing, to measure the final intraoperative joint gaps. The second method was to introduce the device after the tibial resection in a TF workflow to measure gaps prior to the femoral resection. These data are then fed into a predictive gap balancing algorithm and used to plan the femoral resections to achieve equal mediolateral (ML) gaps at 10° and 90° flexion as a primary objective and equal gaps throughout flexion if possible. In both methods, data from the BalanceBot can be blinded from the surgeon to allow the impact of a change in workflow and introduction of new technology to be assessed independently. The study population was split into 4 groups detailed below and in Figure 2:

1) FF blind: FF measured resection workflow where the femur and tibia are cut independently (±1.5° from neutral) and then soft tissue balancing is performed with conventional trials in situ using ligament releases or bone recuts. The BalanceBot was then used to capture final balance achieved immediately before cementing with the surgeon blinded to the BalanceBot data capture.
2) FF unblind: FF measured resection workflow in which the surgeon could see the BalanceBot data after initial femoral and tibial cuts (±1.5° from neutral) and perform recuts or releases before recording a final joint balance with the BalanceBot.
3) TF blind: TF gap balancing workflow in which the surgeon executed a neutral tibial resection (±1.5° from neutral) then used spacer blocks to assess prefemoral resection gaps and conventional trials for soft tissue balancing after all resections. The BalanceBot was then used to capture final balance achieved immediately before cementing with the surgeon blinded to the BalanceBot data capture (as in group 1).
4) TF unblind: TF predictive balancing workflow in which prefemoral resection joint gaps were obtained after the tibial resection (±1.5° from neutral) with the BalanceBot and used for planning the femoral resections using a predictive gap algorithm (4° internal rotation to 8° external rotation and ±5° femoral coronal alignment), after which final joint gaps were recaptured and observed by the surgeon.

**Figure 3:**
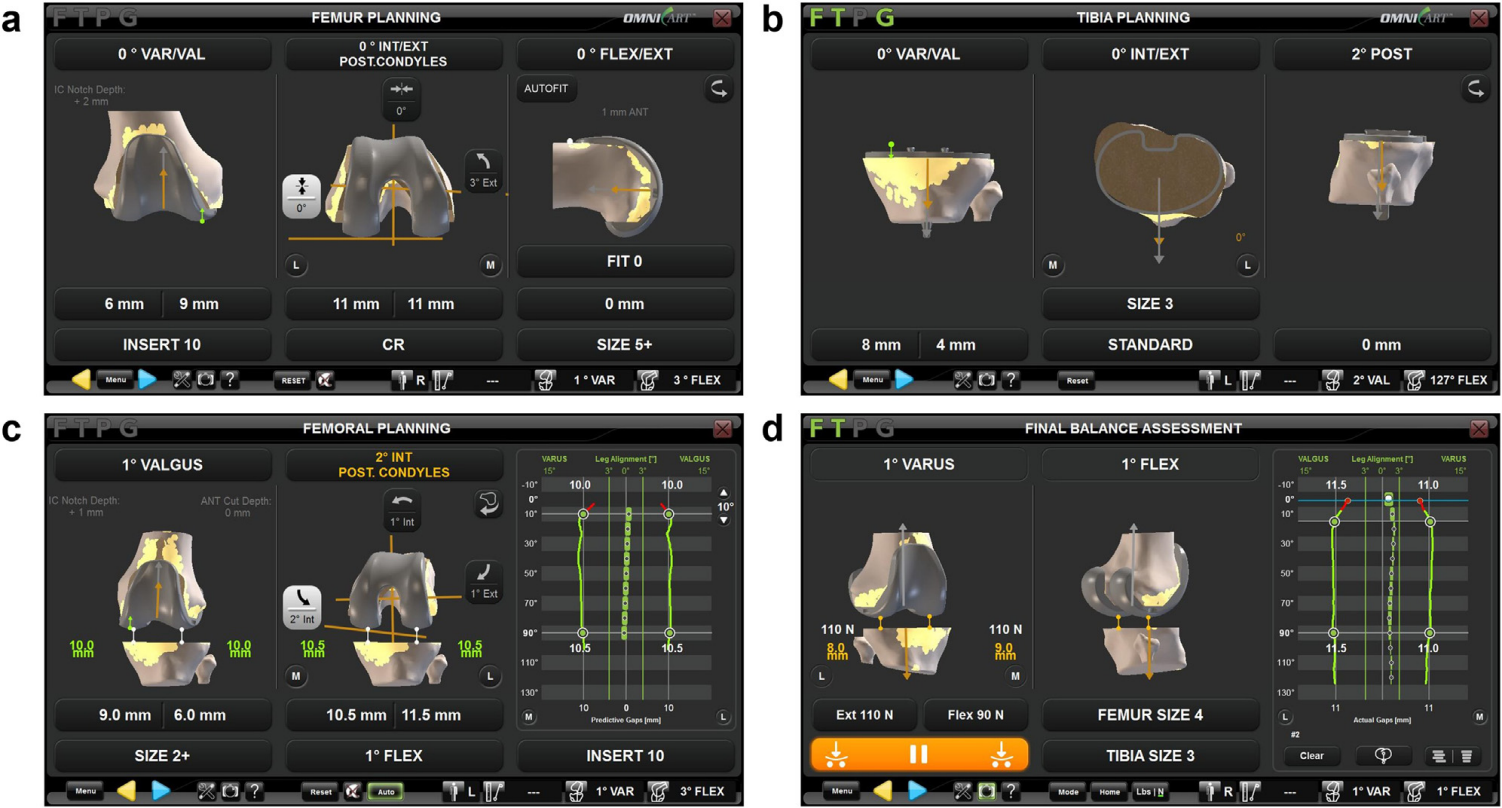
Examples of OMNIBotics. (a) Femur resection planning screen in FF workflow. (b) Tibia resection planning screen in either FF or TF workflow. (c) Femoral component planning based on joint gaps measured in the TF unblind group. (d) Final gap joint balance data capture. Green and blue lines on right indicate measured gaps throughout flexion; individual gap values at 10° and 90° indicated in white numbers.

Participants underwent posterior cruciate ligament-sacrificing TKA, and the surgeon targeted equal gaps mediolaterally in flexion and extension. Corin Apex (Corin Ltd, UK) components were used with an ultracongruent tibial insert and cruciate retaining femoral component. Examples of the FF and TF resection planning screens, post-tibia-resection balance, and final data capture screen showing the component planning and gap data captured in all groups are shown in Figure 3.

### Patient cohort and outcome scores

One hundred six patients were prospectively enrolled. The demographics for the whole cohort and subgroups are shown in Table 1; no differences were identified between groups. Knee Injury and Osteoarthritis Outcome Score (KOOS) and UCLA Activity score (UCLA) were obtained preoperatively and at 3 months, 6 months, and 1 year postoperatively.

**Table 1:** Demographics of whole cohort and subgroups, *P* value shown for Kruskal-Wallis test (age, BMI), and Chi-square test (gender).

Demographic	Whole cohort	Group 1	Group 2	Group 3	Group 4	*P* value
N	106	22	38	22	24	
Age (y)	69.0 ± 9.1	66.3 ± 8.9	70.9 ± 7.7	70.6 ± 8.4	66.8 ± 11.4	.141
Gender (%F)	60	68	53	55	71	.329
BMI (kg/m^2^)	30.7 ± 3.7	31.1 ± 3.4	31.4 ± 3.6	29.3 ± 3.5	30.7 ± 4.2	.213
Coronal deformity (° varus)	5.3 ± 5.4	5.6 ± 5.4	4.6 ± 6.4	5.0 ± 4.2	6.3 ± 3.7	.720
Flexion contracture (°)	3.8 ± 5.2	3.7 ± 5.9	4.1 ± 5.1	4.5 ± 5.0	3.4 ± 5.3	.966

BMI, body mass index; F, female.

### Analysis

To address the primary aim of a change in balance, a power analysis was performed. Performing a 2-sample, 2-sided equality power analysis with (1-β) = 0.8, α = 0.05, a sampling ratio of 1:1, a standard deviation of 1.0 mm [5], and a mean balance difference of 1 mm requires a minimum of 16 cases. A further post-hoc power analysis was performed once all outcome scores were captured to determine the minimum difference in KOOS scores that could be detected by this study. By performing a 2-sample, 2-sided equality power analysis with (1-β) = 0.8, α = 0.05, a KOOS score standard deviation of 15 points, and sampling ratio of 1:1, this study is powered to determine a 13-point difference in outcome.

ML gap difference is defined as the medial gap minus the lateral gap at both flexion angles. The actual and absolute values were recorded and compared. Wilcoxon rank-sum nonparametric t-tests were used to compare joint gaps between groups. A further analysis was performed to determine the average frequency of a knee being classified as balanced using a threshold of 2-mm imbalance [6,7]. These groups were compared using a Chi-square test. Outcome comparisons were performed using 1-way analysis of variance tests. In all cases, significance was determined with a *P* value ≤ .05. The analysis was performed in R4.0.4.

## Results

### ML imbalance

**Figure 4:**
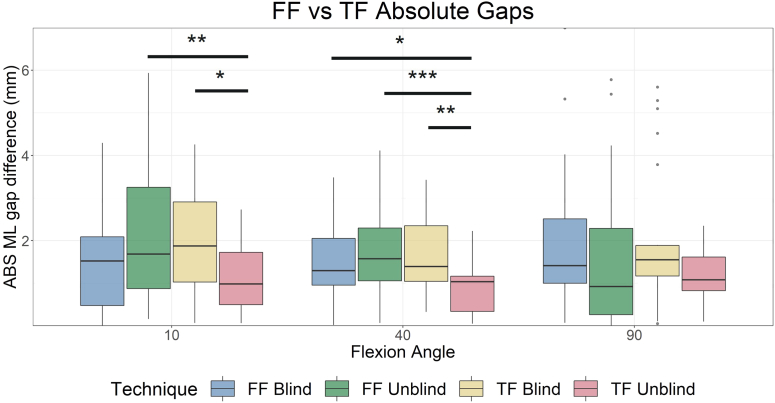
Comparison of absolute value of the mediolateral (ML) imbalance between groups. Significant differences are observed in which the tibia-first (4) group reports reduced joint imbalance compared to all other groups in midflexion and the femur-first (2) and tibia-first blind (3) groups in extension. ABS, absolute value. ∗*P* < .05, ∗∗*P* < .01, ∗∗∗*P* < .001.

TF unblind reported significantly reduced absolute ML imbalance in extension compared to FF unblind (1.1 ± 0.8 mm vs 2.1 ± 1.6 mm, *P* = .006) and TF blind (1.9 ± 1.2 mm, *P* = .045) and in midflexion compared to all groups (TF unblind: 1.0 ± 0.6 mm vs FF blind: 1.5 ± 0.9 mm, *P* = .031; FF unblind: 1.7 ± 1.0 mm, *P* = .001; TF blind: 1.6 ± 1.0 mm, *P* = .009), see Figure 4. No difference was observed between absolute balance in flexion.

**Figure 5:**
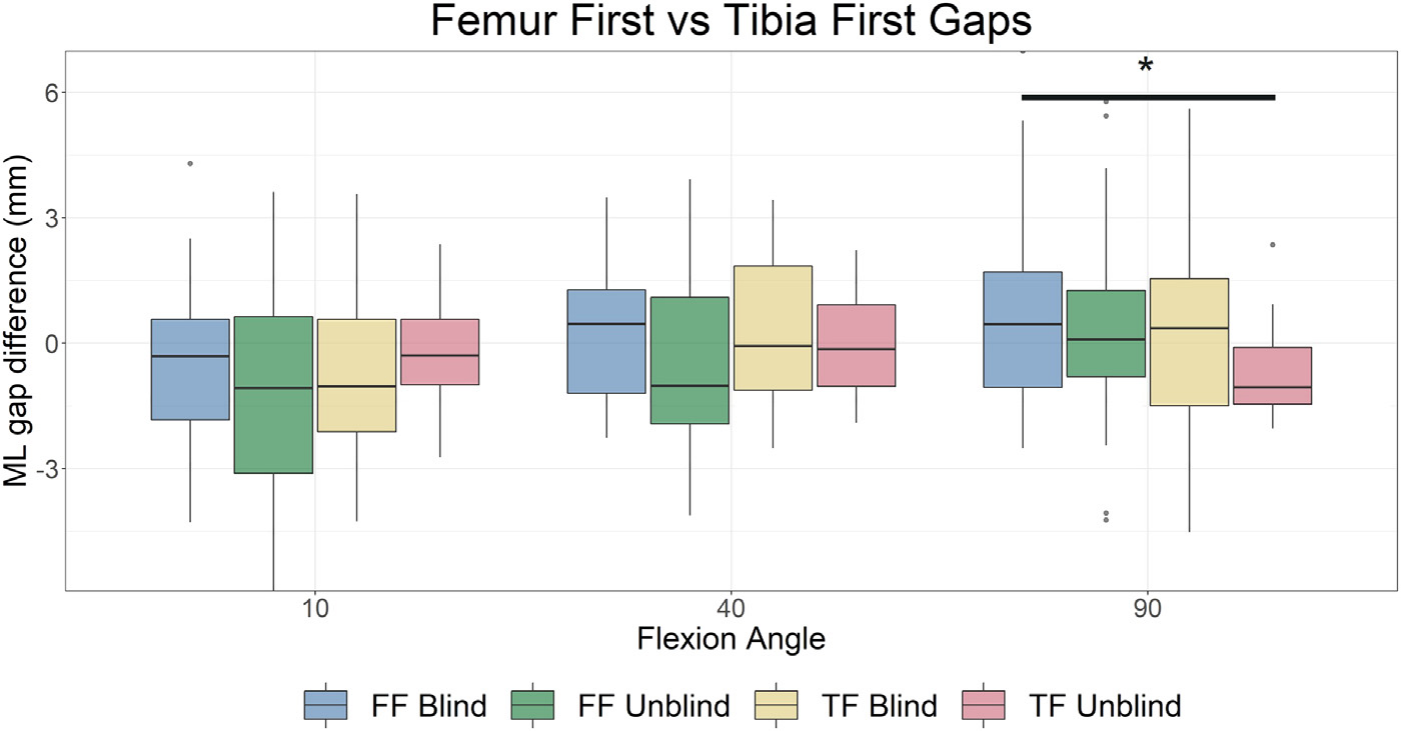
Comparison of mediolateral (ML) imbalance between groups. Average values for all groups are close to zero indicating that knees are as likely to be imbalanced favoring the medial side as favoring the lateral side. A significant difference was observed between the femur first-blind group and tibia-first unblind group in which the tibia-first group showed on average a tighter medial side than lateral side compared to the femur-first blind group. ∗*P* < .05.

Furthermore, TF unblind reported a relatively tighter medial compartment than lateral compartment compared to FF blind in flexion (−0.7 ± 1.2 vs 0.8 ± 2.5, *P* = .029) (Fig. 5); however, both means were within 1 mm of equal balance. No other ML balance differences were found between any groups at any flexion angle.

### Balance variance

**Figure 6:**
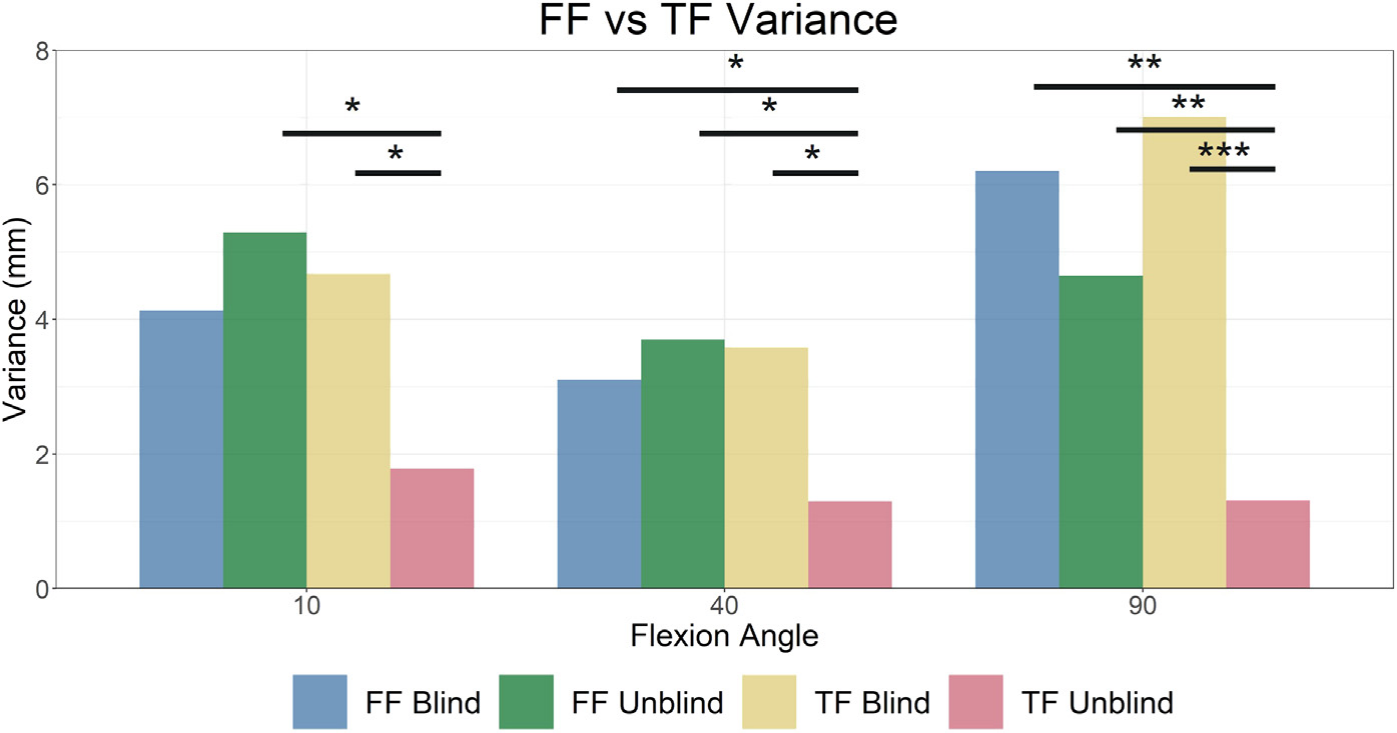
Comparison of variance in mediolateral (ML) joint balance. Significant differences are observed in which the tibia-first unblind group reports reduced variance compared to all other groups in flexion and midflexion and reduced variance compared to the femur-first unblind and tibia-first blind groups in extension. ∗*P* < .05, ∗∗*P* < .01, ∗∗∗*P* < .001.

Reduced balance variance was found throughout flexion in the TF unblind group compared to all other groups with the exception of FF blind in extension (Fig. 6). The TF unblind group reported variances of ≤1.8 mm, up to 3.9-fold reduced variance compared to the other groups.

### Balance frequency

**Figure 7:**
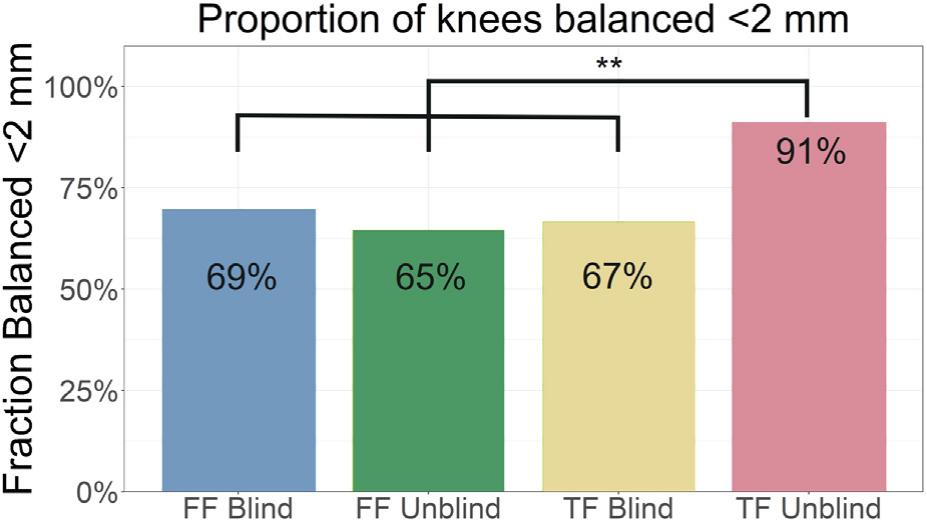
Comparison of fraction of knees balanced ≤2 mm averaged throughout the range of motion. The tibia-first group reports a significantly improved fraction compared to all other groups (91% vs 65%-69%). ∗∗*P* < .01.

A threshold of 2 mm was applied to each balance measurement in extension, midflexion, and flexion across the 4 groups, which was then averaged throughout flexion. The TF unblind group reported a greater proportion of joints balanced to within 2 mm averaged throughout flexion compared to all other groups (*P* ≤ .006) (Fig. 7). TF blind, FF blind, and FF unblind were not significantly different from each other.

### Early outcomes

No differences in KOOS or UCLA scores were observed between the 4 groups preoperatively or at 3, 6, or 12 months postoperatively, see Table 2.

**Table 2:** Early KOOS and UCLA outcome data across all groups.

Time	FF blind	FF unblind	TF blind	TF unblind	*P* value
KOOS pain					
Preop	43.8 ± 16.8	48.6 ± 14.5	49.1 ± 20.1	44.7 ± 16.2	.583
3M	78.9 ± 14.7	77.2 ± 16.2	79.9 ± 15.5	80.9 ± 16.3	.828
6M	84.4 ± 11.6	80.4 ± 17.3	88.9 ± 11.5	82.4 ± 18.6	.275
1Y	84.6 ± 12.1	88.7 ± 12.1	87.3 ± 13.2	84.9 ± 17.8	.677
KOOS symptoms					
Preop	52.2 ± 21.2	51.6 ± 17.6	51.8 ± 20.7	49.9 ± 19.9	.978
3M	76.6 ± 15.5	69.5 ± 17.8	72.8 ± 11.2	75.2 ± 13.6	.328
6M	81.0 ± 13.0	78.5 ± 11.8	79.3 ± 11.5	77.2 ± 19.0	.854
1Y	85.7 ± 8.8	84.1 ± 12.1	80.3 ± 12.6	81.4 ± 15.4	.471
KOOS ADL					
Preop	48.3 ± 15.8	51.9 ± 14.6	48.9 ± 17.6	48.7 ± 11.8	.754
3M	84.7 ± 12.9	80.5 ± 14.4	79.3 ± 13.8	84.6 ± 14.1	.442
6M	87.1 ± 10.6	78.8 ± 20.6	89.7 ± 9.2	85.2 ± 16.9	.082
1Y	88.0 ± 9.3	87.8 ± 14.5	88.9 ± 10.9	85.8 ± 17.7	.910
KOOS QOL					
Preop	21.7 ± 17.2	25.7 ± 16.6	22.4 ± 17.4	19.3 ± 18.3	.548
3M	65.3 ± 14.5	58.3 ± 20.3	65.8 ± 19.8	66.2 ± 16.0	.274
6M	67.4 ± 20.8	64.9 ± 22.1	71.9 ± 20.2	69.6 ± 22.8	.685
1Y	72.7 ± 18.5	75.4 ± 18.3	77.7 ± 20.4	75.0 ± 26.0	.899
KOOS sports					
Preop	20.5 ± 24.7	20.3 ± 17.5	14.3 ± 15.0	16.7 ± 15.1	.578
3M	59.8 ± 24.7	61.9 ± 29.7	53.8 ± 24.0	53.6 ± 28.2	.598
6M	63.9 ± 21.0	61.8 ± 26.9	68.5 ± 19.7	60.4 ± 27.2	.719
1Y	66.1 ± 21.1	70.7 ± 24.1	71.2 ± 23.0	68.2 ± 27.5	.887
UCLA activity					
Preop	3.86 ± 1.11	4.68 ± 1.56	4.09 ± 1.27	4.79 ± 1.98	.103
3M	4.60 ± 1.67	4.89 ± 1.61	4.76 ± 1.51	4.95 ± 1.43	.881
6M	4.94 ± 1.26	5.08 ± 1.73	5.75 ± 1.33	4.91 ± 1.59	.273
1Y	4.84 ± 1.3	5.85 ± 1.88	5.50 ± 2.01	5.05 ± 1.31	.159

*P* value for analysis of variance test between groups shown in the last column. All *P* values are greater than .05.

3M, 3 months; 6M, 6 months; 1Y, 1 year.

## Discussion

The main finding of this study is that the TF unblind group, utilizing predictive balancing, achieved reduced absolute ML imbalance in extension and midflexion, reduced variance in balance throughout flexion, and improved the proportion of knees balanced mediolaterally ≤2 mm averaged throughout the range of motion compared with the TF blind group without predictive balancing and the FF groups both with and without access to joint balance data. This study confirms our first hypothesis and indicates that using a TF approach with a digital joint balancing device that can predict postoperative joint balance, surgeons can better achieve their target balance.

No difference was observed in either KOOS or UCLA outcomes throughout the first year between any of the 4 groups in opposition to our second hypothesis. Previous work by Keggi et al. [8] and Wakelin et al. [9] have shown that similarly balanced knees reported improved KOOS scores in the first year after TKA; however, the improvements were less than what this study was powered to detect. Although the current study was powered for score differences of 13 points, the whole-cohort average KOOS pain score at 1 year is 86.8 ± 13.6 points indicating that group 4 would need an average score of 99.8 to detect a difference. Additionally, previous literature has reported conflicting evidence on the impact of digital joint balance tools to improve outcomes. Golladay et al. [10], Chow and Breslauer [11], and Gustke et al. [12] have found improved outcomes with the use of joint-pressure-sensing devices; however, MacDessi et al. [13] and Song et al. [14] did not demonstrate an improvement. This may indicate that although joint gap and pressure-sensing devices can improve surgeon accuracy, the ideal joint balance on a patient-specific level has not yet been identified.

Recent work by Vigdorchik et al. [1] has shown alignment has no impact on outcome, yet soft-tissue release may negatively impact KOOS scores out to 2 years postoperatively. The impact of workflow on resection angle and frequency of soft-tissue release was not investigated here; however, our results suggest a TF approach with a predictive balancing tool allowing a target balance to be achieved more accurately may help reduce the need for soft-tissue releases and improve outcomes.

No significant balance difference was observed between the 2 FF groups and the TF blind group, indicating that the full benefit of balance technology was not realized when used in a reactive way (ie, after all resections). In these groups, soft-tissue releases were required to achieve final balance, which remains a manual and difficult process without assistive technology [15]. The surgeon in this study has over 35 years of FF experience and balancing after all resections, and as such, the additional final balance data did not improve balance results. In addition, the change in workflow from FF to TF without access to the balance data also did not result in a significant change in balance as the final balancing step remains the same. The results therefore suggest that a surgeon can more precisely and easily achieve a target balance using a predictive balance workflow, adjusting the femoral component position during planning and executing with high accuracy [16,17], rather than performing soft-tissue releases or recutting bone.

This study has several limitations. A single surgeon at a single site utilizing a single implant and robotic system was used here. While this may limit the generalizability of these results, it is also a strength as several confounding factors would arise with multiple surgeons, sites, implants, and robotic platforms. Furthermore, there are no significant differences in demographics including coronal and sagittal preoperative deformity, further reducing confounders. Neutral joint balance was assumed to be the optimal balance target in flexion and extension; however, it is unclear if this is true. Previous studies have shown improved outcomes with asymmetric soft-tissue balance targets with varying flexion [9]. The current study, however, shows an improved ability of a surgeon to achieve a predefined target. The KOOS and UCLA scores reported in this study are early outcomes only. Further follow-up at 5 and 10 years, including survivorship, are required to better understand the long-term impact of the technology. The sample size of this study is underpowered to adequately detect the minimum clinically important difference of the KOOS subscore (8 points) [18]. Additional power would elucidate further the impact of more accurately achieving a predefined balance target below the minimum clinically important difference. The BalanceBot applied a force of 70-90 N to the joint. It is unknown whether this force produces the optimal joint tension postoperatively across the whole population, or whether this joint tension is patient-specific. Finally, when executing an FF workflow, a complete medial collateral ligament soft-tissue slide release (as necessary in certain severe fixed varus deformity cases) may be performed. When this occurs, the final intraoperative BalanceBot values are captured before the medial collateral ligament has healed properly and may not accurately represent the postoperative soft-tissue balance. This patient subset may exhibit a pseudo falsely opened medial gap until the release has healed down into its new position providing the necessary stability for that FF alignment.

## Conclusions

A TF approach using a predictive digital balancing device allowed a target joint balance to be more accurately and consistently achieved than in a FF approach or TF approach without predictive balancing (ie, reactive balancing). Short-term patient outcomes showed no significant difference between groups and require more investigation to define optimal joint balance targets.

## Funding

Corin USA provided research support for this study.

## Conflicts of interest

Dr. Plaskos is a paid employee of Corin USA and has stocks in Corin USA. Dr. Wakelin is a paid employee of Corin USA. Dr. Koenig receives royalties from, gives paid presentations for, is a paid consultant for, and receives research support as a principal investigator from Corin USA. Dr. Shalhoub is a paid employee of Corin USA and has stocks in Corin USA. Dr. Passano declares no potential conflicts of interest.

For full disclosure statements refer to https://doi.org/10.1016/j.artd.2022.06.020.

## Appendix A Supplementary data

Conflict of Interest Statement for Plaskos

Conflict of Interest Statement for Wakelin

Conflict of Interest Statement for Koenig

Conflict of Interest Statement for Passano

Conflict of Interest Statement for Shalhoub
