# Supplementary material for: Impact of a Digital Balancing Tool on Femur and Tibial First Total Knee Arthroplasty: A Prospective Nonrandomized Controlled Trial
Source: Arthroplast Today. 2022 Sep 22;17:172–8. doi: 10.1016/j.artd.2022.06.020 (PMC9519394; doi:10.1016/j.artd.2022.06.020)
Supplement: Conflict of Interest Statement for Wakelin [file mmc2.docx]

# CONFLICT OF INTEREST STATEMENT

***American Association of Hip and Knee Surgeons***

(Adopted from the American Academy of Orthopaedic Surgeons disclosure statement)

The following form **must be filled out completely and submitted by each author (example, 6 authors, 6 forms).**

**All items require a response. If there is no relevant disclosure for a given item, enter "*None*.”**

Impact of a Digital Balancing Tool on Femur and Tibial First Total Knee Arthroplasty: A Prospective Non-Randomized Controlled Trial

Manuscript Title

1. Royalties from a company or supplier (The following conflicts were disclosed)

None

2. Speakers bureau/paid presentations for a company or supplier (The following conflicts were disclosed)

None

3A. Paid employee for a company or supplier (The following conflicts were disclosed)

Paid employee of Corin USA

3B. Paid consultant for a company or supplier (The following conflicts were disclosed)

None

3C. Unpaid consultants for a company or supplier (The following conflicts were disclosed)

None

4. Stock or stock options in a company or supplier (The following conflicts were disclosed)

None

5. Research support from a company or supplier as a Principal Investigator (The following conflicts were disclosed)

None

6. Other financial or material support from a company or supplier (The following conflicts were disclosed)

None

7. Royalties, financial or material support from publishers (The following conflicts were disclosed)

None

8. Medical/Orthopaedic publications editorial/governing board (The following conflicts were disclosed)

None

9. Board member/committee appointments for a society (The following conflicts were disclosed)

None

**Each author must sign AND print or type his/her name, date and submit a separate form**

In addition, one BLINDED Conflict of Interest form (no author names used) should be submitted per manuscript with all author disclosures.

Edgar Wakelin
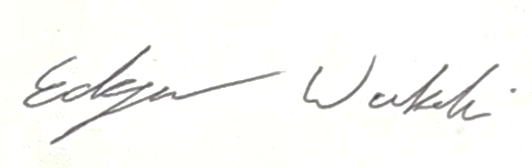
 02/03/2021

Author Name (Print or Type) Author Signature Date
